# A Novel Method for Fabricating the Undulating Structures at Dermal—Epidermal Junction by Composite Molding Process

**DOI:** 10.3390/jfb15040102

**Published:** 2024-04-15

**Authors:** Hao Qiao, Chuang Gao, Chunxiang Lu, Huazhen Liu, Yi Zhang, Aoxiang Jin, Qiqi Dai, Shihmo Yang, Bing Zhang, Yuanyuan Liu

**Affiliations:** 1 School of Mechatronic Engineering and Automation, Shanghai University, Shanghai 200444, China; 1787758318@shu.edu.cn (H.Q.); gaochuang128634@163.com (C.G.); cxlu@shu.edu.cn (C.L.); zhangyishu@shu.edu.cn (Y.Z.); 2845879177@shu.edu.cn (A.J.); smyang@shu.edu.cn (S.Y.); bingzhang84@shu.edu.cn (B.Z.); 2 School of Medicine, Shanghai University, Shanghai 200444, China; lesyinz@163.com (H.L.); qqidia@163.com (Q.D.)

**Keywords:** PLGA-PCL nanofibers, microstructure, electrospinning, 3D printing, micro-imprinting

## Abstract

The dermal–epidermal junction (DEJ), located between the dermal–epidermal layers in human skin tissue, plays a significant role in its function. However, the limitations of biomaterial properties and microstructure fabrication methods mean that most current tissue engineered skin models do not consider the existence of DEJ. In this study, a nanofiber membrane that simulates the fluctuating structure of skin DEJ was prepared by the composite molding process. Electrospinning is a technique for the production of nanofibers, which can customize the physical and biological properties of biomaterials. At present, electrospinning technology is widely used in the simulation of customized natural skin DEJ. In this study, four different concentration ratios of poly (lactic-co-glycolic acid) (PLGA) and polycaprolactone (PCL) nanofiber membranes were prepared based on electrospinning technology. We selected a 15%PLGA + 5%PCL nanofiber membrane with mechanical properties, dimensional stability, hydrophilicity, and biocompatibility after physical properties and biological characterization. Then, the array-based microstructure model was prepared by three-dimensional (3D) printing. Subsequently, the microstructure was created on a 15%PLGA + 5%PCL membrane by the micro-imprinting process. Finally, the cell proliferation and live/dead tests of keratinocytes (HaCaTs) and fibroblasts (HSFs) were measured on the microstructural membrane and flat membrane. The results showed that 15%PLGA + 5%PCL microstructure membrane was more beneficial to promote the adhesion and proliferation of HaCaTs and HSFs than a flat membrane.

## 1. Introduction

DEJ, as a specific form of the extracellular matrix (ECM), is widely distributed in human skin, kidney, digestive tract, lung, and other tissues [1]. Among these are the DEJ of skin tissues, which exist at the junction of the two layers of the epidermal–dermal structure of the skin and act as a barrier between the epidermis and the dermis [2]. The composition, structure, and morphology of DEJ have an important influence on skin tissue cells. Functionally, the DEJ can provide physical support to cells and regulate the maintenance of signs between cells and surrounding tissues [3,4]. There are up-and-down undulating microstructures on the DEJ called rete ridges [5,6]. The rete ridge structure increases the surface area of the epidermal–dermal layer of skin tissues and improves the mechanical strength of the skin [7,8]. Therefore, the DEJ with undulating microstructures can better improve the bionic level of tissue engineered skin.

Current artificial skin substitutes developed in the field of tissue engineering and regenerative medicine for transplantation often lack the microstructure at DEJ. This deficiency makes it challenging for artificial skin to fully replace human skin tissue. In a previous study, collagen–glycosaminoglycan materials were utilized to replicate a skin model consisting of rete ridge structures, which demonstrated a capacity to enhance the proliferation and differentiation of keratinocytes and that the proliferative capacity of HaCaTs increased as the depth of the rete ridge structure increased and the width of the channel decreased [9]. In another report, PCL nanofiber membranes with rete ridge microstructures were prepared by combining electrospinning with a microstructure receiver. By comparing the co-culture of human dermal keratinocytes (HDK) on normal PCL membranes with microstructures, the results showed that rete ridge microstructures had a positive effect on the metabolic activity of HDK [10]. Therefore, the selection of appropriate biomaterials for preparing nanofiber membranes with rete ridges is crucial.

PLGA, as a polymeric material, has good biocompatibility, biodegradability, and high mechanical strength [11,12,13,14]. Therefore, PLGA is widely used in wound repair, drug release, and implantable bio-scaffolds [15,16,17]. However, PLGA nanofibers have low elongation and brittleness. In addition, the stability of PLGA nanofibers is affected by temperature and the liquid environment. To solve the shrinkage problem of PLGA nanofibers, another polymer component is usually added to the electrospinning solution to improve the stability of the nanofibers [18,19,20]. 

PCL is a biodegradable synthetic polymer with a low melting point, good solubility, biocompatibility, stability, and mechanical properties. These properties make it a popular material in tissue engineering [21,22,23]. Furthermore, PCL has been approved by the U.S. Food and Drug Administration (FDA) [24,25]. However, PCL is a hydrophobic material lacking cell adhesion sites on its surface, preventing cell adhesion, migration, proliferation, and differentiation [15,21,26]. Previous studies have shown that the limited cell adhesion and low biodegradability of PCL may be overcome by incorporating other biomaterials [27,28].

In our previous study, skin models with rete ridges were fabricated on PCL nanofiber membranes using 3D printing. Biological experiments such as histological and immunofluorescence testing were performed on the microstructural skin model after a period of air–liquid interface culture. The results showed that the rete ridge structure played a positive role in cell proliferation, viability, and differentiation [29]. However, extrusion-based 3D printing still makes it difficult to directly shape microstructures with a certain curvature. 

**Figure 1:**
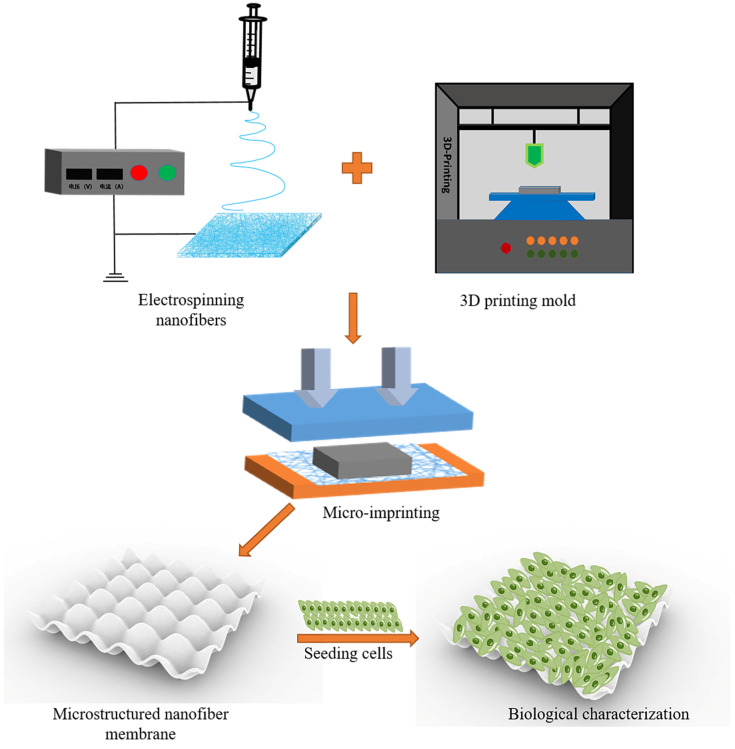
Preparation process of microstructured nanofiber membrane.

In this context, this paper innovatively combined electrospinning and micro-imprinting processes to create a nanofiber membrane that simulates the fluctuating structure of natural skin DEJ. In this study, four kinds of PLGA-PCL membranes were prepared by adding different proportions of PCL materials to a PLGA electrospinning solution. Then, PLGA-PCL membranes with comprehensive properties were selected through Fourier transform infrared spectroscopy (FTIR) testing, micro-morphology, mechanical properties, dimensional stability, hydrophilicity, and biocompatibility of different membranes. At the same time, microstructural molds with a diameter, depth, and spacing of 200 μm were fabricated using a light-curing 3D printer. Finally, PLGA-PCL microstructural membranes were prepared by the micro-imprinting process. The preparation process is shown in Figure 1.

## 2. Materials and Methods

### 2.1. Materials

PLGA (lactide/glycolide ratio of 50:50, M_W_ = 100,000 Da), PCL (M_W_ = 80,000 Da), and 1,1,1,3,3,3-Hexafluoroisopropanol (HFIP) solution were purchased from Aladdin (Shanghai, China). Phosphate-buffered saline (PBS), Dulbecco’s modified Eagle’s medium (DMEM), fetal bovine serum (FBS), and penicillin/streptomycin (P/S) were bought from (Shanghai, China). Cell counting-kit-8 (CCK8) reagent was acquired from APExBIO (Beijing, China). Live/dead staining reagents were obtained from Solarbio (Shanghai, China).

### 2.2. Preparation of Electrospinning Solution

The electrospinning solution was prepared by dissolving PLGA and PCL in HFIP solution. Four PLGA-PCL polymer electrospinning solutions with concentrations of 15%, 15% + 3%, 15% + 5%, and 15% + 7% (*w*/*v*) were formulated depending on the PCL content. The four different concentration solutions were then stirred at 180 rpm for 5 h at room temperature until the PLGA-PCL solutions were completely mixed.

### 2.3. Electrospinning PLGA-PCL Nanofiber Membrane

The well-stirred PLGA-PCL solution was injected into a 10 mL loaded 21 G metal needle syringe. The syringe was mounted on the electrospinning platform. The positive pole of the high-voltage power supply was connected to the metal needle, and the negative pole was connected to the electrospinning receiving platform. A vertically adjustable metal frame structure with a flat surface was utilized as the electrospinning collector, with dimensions of 10 cm × 10 cm. The parameters of electrospinning were set as follows: The voltage was 11 KV, the flow rate of the microinjection pump was 2 mL/h, and the distance between the metal needle and the electrospinning receiving platform was 15 cm. The electrospinning process was concluded when the thickness of the membrane reached 80 μm. The size of the membrane obtained after electrospinning is the same as the surface size of the receiver. Subsequently, the obtained membrane was subjected to oven-drying. The electrospinning process was conducted at room temperature with a humidity range of 35–45%.

### 2.4. Physical Characteristics of PLGA-PCL Nanofiber Membranes

#### 2.4.1. Scanning Electron Microscope (SEM)

The PLGA-PCL nanofiber membranes with different concentration ratios were fixed on an aluminum block. The surface of the nanofiber membrane was sprayed with gold using a high-vacuum ion sputter coater (ACE-600, Leica, Shanghai, China). Then the aluminum block was placed on the sample stage. Finally, the microstructure of the surface of the gold-plated samples was observed and imaged by a field emission scanning electron microscope (7100F) (JEOL LTD, Tokyo, Japan). Furthermore, image software was used to measure the diameters of at least 50 nanofibers from the SEM images.

#### 2.4.2. FTIR Test

Chemical compositions of nanofiber membranes were evaluated using a spectrometer instrument (NicoletiS10, Thermo Fisher, Waltham, MA, USA). Wavelength range was set to 500 to 4000 cm^−1^ with a resolution of 4 cm^−1^. 

#### 2.4.3. Shrinkage Test

The four PLGA-PCL nanofiber membranes were each cut into rectangles of fixed size. The initial area of the membranes was Va. The degree of shrinkage of the four different membranes was calculated after the prepared nanofiber membranes were immersed in DMEM solution and placed in an incubator for a period of time. After 0 h, 1 h, and 3 h, the nanofiber membranes were removed from the cell incubator, and the solution on the surface of the membranes was blotted off with filter paper. At this point, the area of the nanofiber membranes was measured again and noted as V_b_. The shrinkage behavior response as a percentage of the area was calculated according to the following Equation (I):

Percentage of area (%) = V_b_/V_a_ × 100% (1)

#### 2.4.4. Hydrophilicity

The hydrophilicity of PLGA-PCL membranes with different concentration ratios was measured using a dynamic contact angle analyzer (BIOLIN Scientific AB, Gothenburg, Sweden). A high-resolution, high-speed camera was used to continuously capture topographical images of the droplets during the test. Finally, the dynamic evolution of the water contact angle of a droplet on a nanofiber membrane was monitored from the moment of initial contact to 10 s thereafter through computer-integrated software measurement. 

#### 2.4.5. Tensile Test

The mechanical properties of electrospinning PLGA-PCL membranes were assessed using the Univert device (CellScale, Ontario, Canada). Initially, four PLGA-PCL nanofiber membranes were sliced into rectangular pieces (10 mm × 25 mm), and their original thickness was gauged. Tensile stress tests were carried out at room temperature. 

### 2.5. Cell Culture and Electrospinning Membranes Preparation

Cells were purchased from Fuheng Biotechnology (Shanghai, China). For the culture of HaCaTs, DMEM high-sugar medium with a mixture of 10% FBS and 1% P/S solution was utilized. HSFs were cultured in a medium containing 10% FBS, 1% P/S solution, and DMEM low-sugar medium. Both HaCaTs and HSFs were changed in the medium once a day. When the cells covered 70–80% of the bottom of the culture dish, the two cell types were passaged in a 1:3 ratio. The prepared nanofiber membranes were cut into 14 mm diameter discs and placed in 24-well plates. Subsequently, the front and back of the nanofiber membrane underwent sterilization with UV light for 1 h [30]. Before inoculating the membranes with cells, the membranes were rinsed three times with PBS solution and immersed in DMEM solution for overnight incubation.

### 2.6. Biological Characterization of PLGA-PCL Membranes

#### 2.6.1. HaCaTs Viability

HaCaTs were seeded onto electrospinning PLGA-PCL membranes at 3 × 10^4^ cells/cm^2^. After 3 days of seeding, the viability of HaCaTs on the membranes was assessed by live/dead staining tests. Experimental solution was prepared by mixing calcein-AM (2 mM) and propidium iodide (1.5 mM) with PBS. The membranes inoculated with HaCaTs were immersed in the live cell staining solution and incubated for 20 min, then the membranes were transferred to the dead cell staining solution and incubated for 5 min. In the end, the membranes were rinsed twice with PBS solution, and the samples were placed under a fluorescence microscope for observation and photography.

#### 2.6.2. HaCaTs Proliferation

The membranes with cells were transferred to the incubator, where they were cultured at 37 °C and 5% CO_2_ while the medium was changed daily. After 1, 2, and 3 days of incubation, the medium was aspirated, and the membranes were washed twice with PBS solution. The work solution was prepared at a volume ratio of CCK-8 reagent: DMEM solution of 10:100 (*v*/*v*). Then, 400 μL of configured CCK-8 work solution was added to the well plates containing the nanofiber membranes. The plates were then incubated in the incubator for three hours. After incubation, 100 μL of solution was removed from each well of the 24-well plate and transferred to a 96-well plate. The optical density (OD) at 450 nm of the solution in each well was measured using a microplate reader (TECAN, Mandry, Switzerland). 

#### 2.6.3. HSFs Viability and Proliferation

HSFs were seeded on four different ratios of PLGA-PCL nanofiber membranes at 1.5 × 10^4^ cells/cm^2^. HSFs were tested for viability and proliferation using the same method as described above for HaCaTs.

### 2.7. Physical and Biological Characterization of Microstructured Membranes 

#### 2.7.1. Preparation of Nanofiber Membranes with Microstructures

Three-dimensional molds were designed using SolidWorks software (2020) and obtained from a standard tessellation language (STL) file. The STL file was then imported into a commercial DLP light-curing 3D printer (From3+, Formlabs, Somerville, MA, USA) to print the microstructure mold. Finally, microstructures on the 3D molds were transferred onto membranes consisting of 15%PLGA + 5%PCL via a micro-imprinting process. The process of micro-imprinting was accomplished by using a pneumatic embosser set at a pressure of 0.2 MPa for 10 min.

#### 2.7.2. Image Evaluation

A 7100F field emission SEM was used to evaluate the microstructural dimensions and surface micromorphology of nanofiber membranes prepared by electrospinning and micro-imprinting.

#### 2.7.3. Cell Viability and Proliferation

The cell seeding density and culture method are the same as those introduced in Section 2.7.1, Section 2.7.2 and Section 2.7.3. Cell viability and proliferation of HaCaTs and HSFs on nanofiber membranes with microstructures were compared with nanofiber flat membranes after 1, 2, and 3 days of culturing. 

### 2.8. Statistical Analysis

The statistical analysis was conducted using Origin software (2021). All the above experiments were performed with at least three replications. The results of all experiments are expressed as the mean ± standard deviation and analyzed by student *t*-test or one-way ANOVA. ** *p* < 0.01 or * *p* < 0.05 was considered statistically significant.

## 3. Result

### 3.1. Physical Characteristics of PLGA-PCL Nanofiber Membranes

#### 3.1.1. SEM

**Figure 2:**
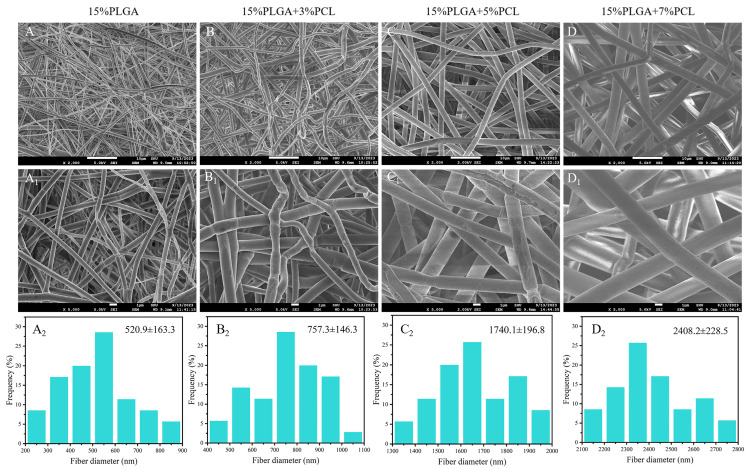
SEM images and diameter distribution of PLGA-PCL nanofiber membranes with different concentration ratios. (**A**–**D**) Micromorphology of PLGA-PCL nanofiber membrane magnified by 2000 times (×2000). (**A_1_**–**D_1_**) The microscopic morphology of PLGA-PCL membrane at 5000 times magnification (×5000). (**A_2_**–**D_2_**) Diameter distribution of PLGA-PCL nanofibers.

As shown in Figure 2A–D,A_1_–D_1_, the surface morphology of nanofiber membranes, produced with four distinct concentration ratios, was presented at various magnifications. The nanofibers produced by the electrospinning process have good diameter uniformity, in addition to the absence of microbead defects on the surface of the nanofiber membranes. The diameter distribution of the nanofibers is shown in Figure 2A_2_–D_2_. The average diameters of the four types of nanofibers were 520.9 ± 163.3 nm, 757.3 ± 146.3 nm, 1740.1 ± 196.8 nm, and 2408.2 ± 228.5 nm, respectively. In the preparation process, except for varying concentrations of the PLGA-PCL electrospinning solution, the environmental (temperature, humidity) and parameters (voltage, flow rate, needle diameter, collector distance) controlling the electrospinning process were kept consistent. Therefore, in the experiment, the properties of electrospinning nanofibers are only related to the electrospinning solution. It was found that as the PCL concentration in the electrospinning solution increased, the viscosity of the solution also increased. The droplet splitting ability decreases as the viscosity of the solution increases, resulting in larger-diameter electrospun nanofiber in a constant voltage during electrospinning [31,32].

#### 3.1.2. FTIR Analysis

**Figure 3:**
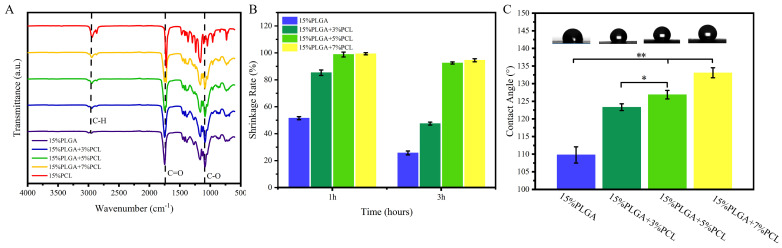
Investigation of the FTIR test, shrinkage characteristics, and hydrophilicity of PLGA-PCL nanofibers. (**A**) Functional group analysis of PLGA-PCL nanofiber membranes using FTIR. (**B**) Shrinkage behavior. (**C**) Hydrophilicity; ** *p* < 0.01, * *p* < 0.05.

The FTIR results of nanofiber membranes are shown in Figure 3A. From the FTIR spectra of PLGA-PCL membranes, characteristic peaks of PLGA can be observed, such as peaks observed between 1750 cm^−1^ and 1760 cm^−1^ representing the stretching vibration of carbonyl groups (C=O) and peaks between 1200 cm^−1^ and 1250 cm^−1^ representing C–O (attributed to ester groups). Furthermore, characteristic peaks of PCL are also observed in composite membranes, with peaks between 2850 cm^−1^ and 3000 cm^−1^ attributed to the stretching vibration of C–H in PCL material. It is proven that the two materials are successfully combined.

#### 3.1.3. Shrinkage Analysis

Stability plays a vital role in tissue engineering scaffolds intended for cell culture and tissue repair [33]. Figure 3B depicts that the nanofiber membrane composed of 15%PLGA exhibited the most significant shrinkage after 3 h of incubation. The area of the nanofiber membrane decreased to 25.69 ± 1.41% of the initial area. Jiang et al. examined the shrinkage behavior of PLGA membranes in PBS solution at 37 °C, revealing that the membranes shrinkage rate reached 80% within 2 h [34]. The membranes consisting of 15%PLGA + 3%PCL, 15%PLGA + 5%PCL, and 15%PLGA + 7%PCL were incubated for 3 h. After incubation, the membranes shrank to 47.48 ± 1.11%, 92.49 ± 0.82%, and 94.53 ± 1.15% of their original area, respectively. The results revealed that the membranes with 15%PLGA + 5%PCL and 15%PLGA + 7%PCL exhibited similar shrinkage characteristics. Current research indicates that PLGA is an amorphous polymer characterized by low crystallinity and inadequate thermal stability. The stability of the PLGA matrix can be effectively enhanced by incorporating an additional polymer material. PCL is a typical crystalline polymer, where the crystalline phases can restrict the mobility of molecules. In addition, integrating PCL nanofibers into PLGA membranes can impede the movement of PLGA molecular chains, thereby enhancing the stability of the PLGA-PCL composite membrane [35]. In this experiment, the stability of the membranes likewise escalates as the concentration of PCL increases. Meanwhile, a related study conducted by Cui et al. indicated that poly(D,L-lactide) (PDLLA) and hydrophilic polyethylene glycol (PEG) combined with PLGA membranes can alleviate the instability of PLGA nanofibers [19].

#### 3.1.4. Hydrophilicity

Hydrophilicity serves as a critical index for assessing the biocompatibility of nanofiber membranes [36]. In this study, the hydrophilicity of PLGA-PCL nanofiber membranes was evaluated using four different concentration ratios, and the findings were illustrated in Figure 3C, which indicated that 15%PLGA nanofiber membranes exhibited the highest hydrophilicity, while the hydrophilicity of electrospinning PLGA-PCL membranes diminished as the concentration of PCL increased in the electrospinning solution. This decline was due to the hydrophobic characteristics of PCL [37,38].

#### 3.1.5. Tensile Analysis

**Figure 4:**
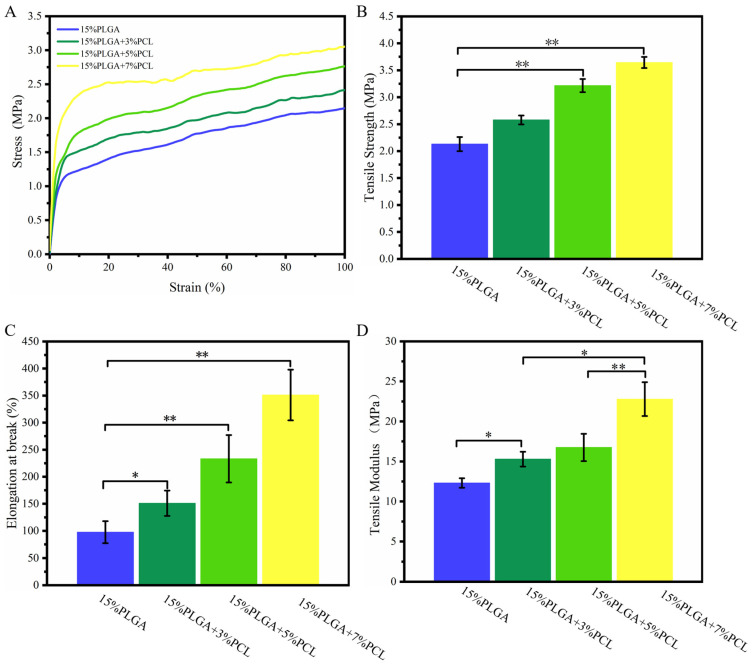
Investigation of the mechanical properties of PLGA-PCL nanofibers. (**A**) Stress–strain curves. (**B**) Tensile strength. (**C**) Elongation at break. (**D**) Tensile modulus; ** *p* < 0.01, * *p* < 0.05.

The mechanical properties of nanofiber membranes significantly affect cell proliferation, migration, and differentiation [39]. Nanofiber membranes of greater stiffness have been found to increase the proliferation and migration of HaCaTs [7,40]. As illustrated in Figure 4A,D, the stress-strain curves of PLGA-PCL nanofiber membranes were utilized to determine their tensile modulus. The findings indicated a correlation between PCL concentration and the tensile modulus of the nanofiber membrane: As the PCL concentration went up, the tensile modulus increased. In addition, we evaluated the tensile strength and fracture elongation of membranes in Figure 4B,C. The addition of PCL to 15%PLGA membranes improved their tensile strength. Specifically, 15%PLGA + 5%PCL and 15%PLGA + 7%PCL exhibited significantly higher tensile strengths with values of 3.21 ± 0.12 MPa and 3.64 ± 0.11 MPa, respectively, compared to 15%PLGA (2.13 ± 0.13 MPa). Meanwhile, 15%PLGA membranes exhibit the lowest elongation at break. The fracture elongation of 15%PLGA + 5%PCL and 15%PLGA + 7%PCL membranes significantly differs from those of 15%PLGA membranes. The reason for this phenomenon is that the increase in PCL concentration leads to an increase in liquid viscosity. With the growing mass concentration of PCL, the number of PCL macromolecular chains as well as the intermolecular forces of PCL molecules increased, and the tensile strength of the electrospinning membrane increased [41]. 

### 3.2. Biological Characterization of PLGA-PCL Nanofiber Membranes 

#### 3.2.1. HaCaTs Viability and Proliferation 

**Figure 5:**
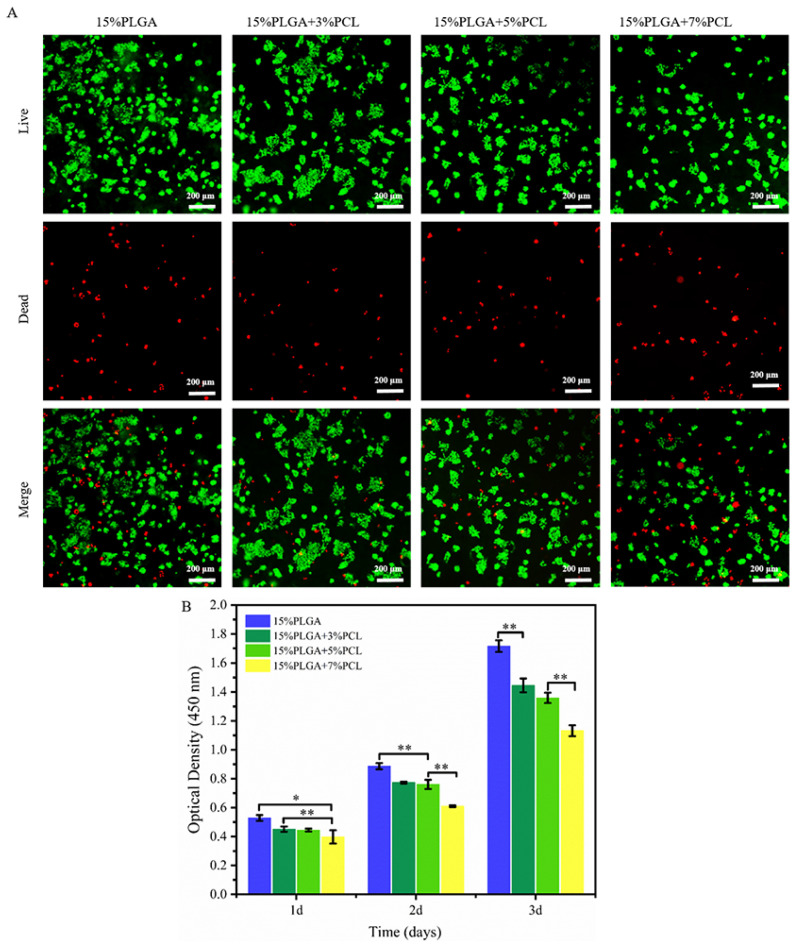
Evaluation of the biocompatibility of PLGA-PCL membranes for HaCaTs. (**A**) Live/dead staining of HaCaTs on nanofiber membranes. (**B**) Quantitative analysis of HaCaTs proliferation; ** *p* < 0.01, * *p* < 0.05.

HaCaTs were cultured on various nanofiber membranes, as depicted in Figure 5A. HaCaTs on nanofiber membranes with different concentration ratios were able to adhere and proliferate. However, the number of viable cells decreased with an increase in PCL concentration within the nanofiber membranes.

Furthermore, the CCK-8 method was used to test the proliferation rate of HaCaTs on various membranes, as demonstrated in Figure 5B. The results indicated that the nanofiber membranes with 15%PLGA had the highest proliferation rate of HaCaTs. Furthermore, 15%PLGA + 7%PCL membranes displayed the lowest proliferation rate among HaCaTs. After 1 day of culture, the proliferation of HaCaTs on the 15%PLGA + 7%PCL membrane was slightly slower than on the 15%PLGA + 5%PCL. However, there were statistically significant differences on the second and third days. The proliferation of HaCaTs on 15%PLGA + 5%PCL membrane was not significantly different from the 15%PLGA + 3%PCL membrane within 3 days of culture. The proliferation rate of HaCaTs decreased with increasing concentrations of PCL within the nanofiber membranes.

#### 3.2.2. HSFs Viability and Proliferation

**Figure 6:**
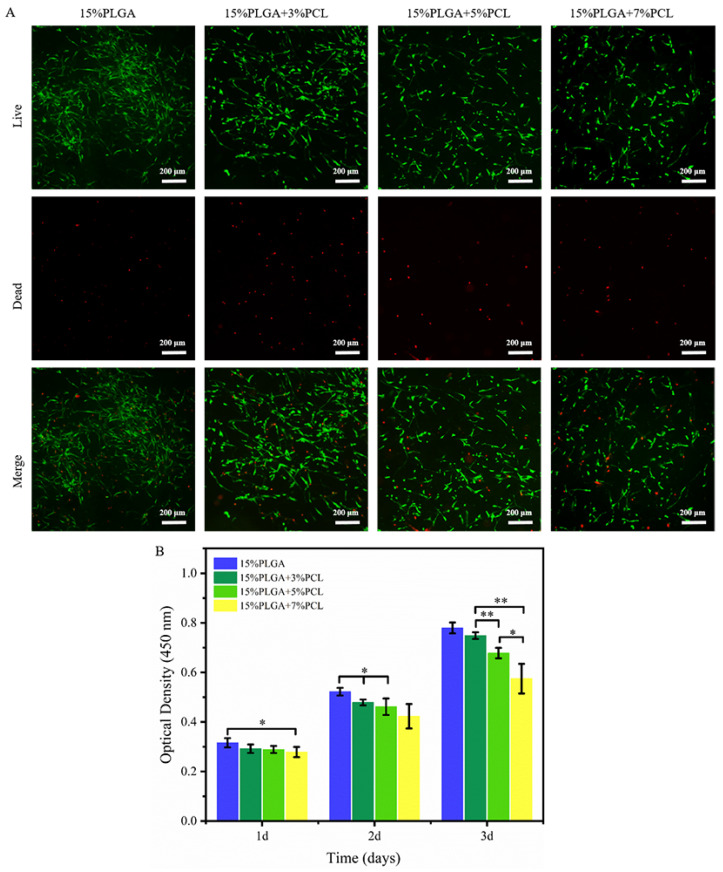
Evaluation of the biocompatibility of PLGA-PCL nanofiber membranes for HSFs. (**A**) Live/dead staining of HSFs. (**B**) Quantitative analysis of HSFs proliferation on various nanofiber membranes; ** *p* < 0.01, * *p* < 0.05.

According to Figure 6A, live/dead staining experiments revealed that the number of viable cells in HSFs declined as the PCL concentration increased in the PLGA-PCL nanofiber membranes. 

The proliferation of HSFs on different membranes is shown in Figure 6B. After 1 day of culture, the proliferation of HSFs on the 15%PLGA + 7%PCL membrane showed a significant difference compared to the 15%PLGA membrane. However, there were no statistically significant differences in the proliferation of HSFs on the 15%PLGA + 5%PCL membrane compared to the 15%PLGA + 3%PCL. There were statistically significant differences in the proliferation of HSFs on the 15%PLGA + 5%PCL nanofiber membrane compared to the 15%PLGA + 3%PCL membranes on the second and third day of culturing. Similar to the proliferation of HaCaTs, the proliferation rate of HSFs was lowest on the 15%PLGA + 7%PCL nanofiber membrane, and the proliferation of HSFs decreased with increasing PCL concentration in the nanofiber membrane. 

Previous studies have demonstrated that the cell proliferation rate decreases as the nanofiber diameter increases [42]. In addition, it has been found that the diameter of nanofibers has different effects on different types of cells, and HaCaTs are more sensitive to the diameter of nanofibers than HSFs [43]. The findings obtained from our experiments align with and support this conclusion. 

### 3.3. Physical and Biological Characterization of Microstructure Membrane

#### 3.3.1. Image Evaluation

**Figure 7:**
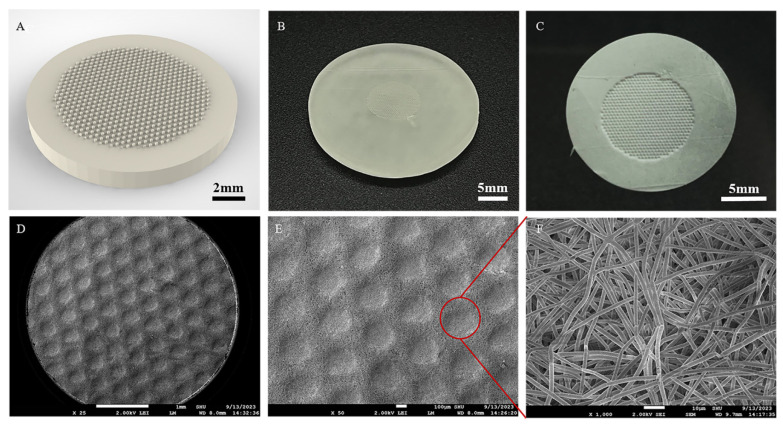
Evaluation of microstructured nanofiber membranes. (**A**) The 3D model of microstructured molds. (**B**) Light-curing printed molds. (**C**) 15%PLGA + 5%PCL membranes prepared. (**D**,**E**) Microstructured nanofiber membranes at different magnifications. (**F**) Micromorphology within the microstructured regions of the nanofibers (×1000).

In summary, 15%PLGA + 5%PCL nanofiber membranes have good mechanical properties, dimensional stability, and biocompatibility. Therefore, we chose 15%PLGA + 5%PCL nanofiber to prepare microstructure membranes, simulate the structure of skin tissue EDJ, and explore the effect of microstructure on skin cells. Figure 7A shows the 3D model of the microstructure mold designed by Solidworks software. The microstructural molds prepared by a light-curing printer are shown in Figure 7B. The surface of the mold was free of defects. The 15%PLGA-5%PCL nanofiber membranes prepared by combining micro-imprinting and electrospinning processes are shown in Figure 7C. The microstructure imprinted on the nanofiber membranes has an obvious outline. The surface micromorphology of 15%PLGA + 5%PCL microstructure membranes is shown in Figure 7D–F. The results showed that the membranes prepared by the micro-imprinting process can still maintain the microstructure. The microstructure size of the prepared nanofiber membranes was 208 ± 13.9 μm after measuring multiple regions of at least 3 membranes with the same microstructure.

#### 3.3.2. HaCaTs Viability and Proliferation

**Figure 8:**
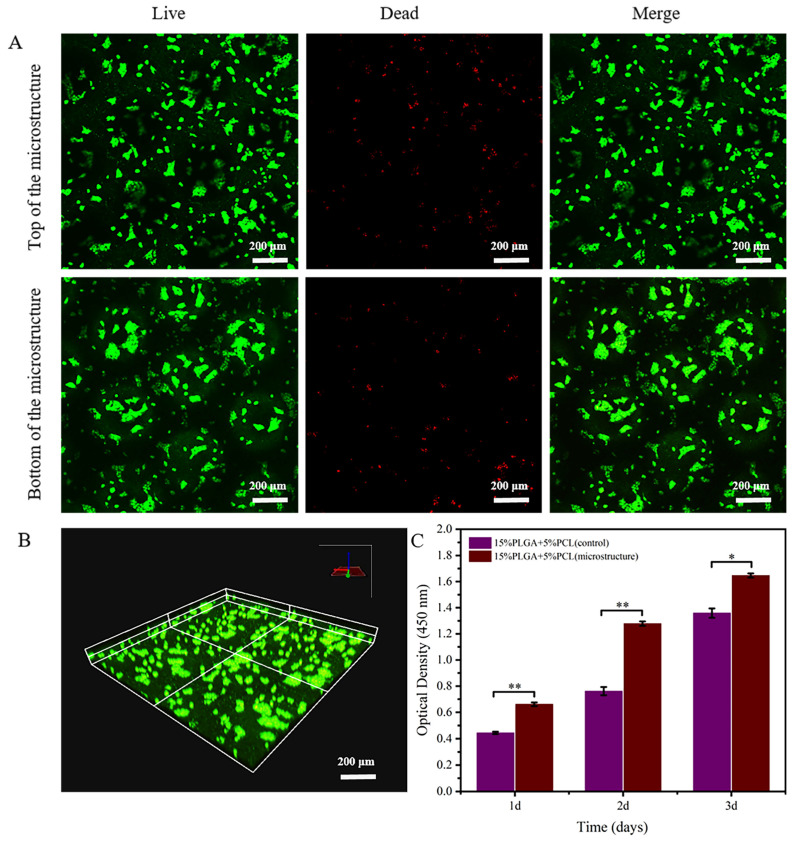
Assessment of biocompatibility of 15%PLGA + 5%PCL microstructure membrane for HaCaTs. (**A**) Live/dead staining of HaCaTs after 3 days of culture. (**B**) Confocal images displaying live/dead staining of HaCaTs. (**C**) Quantitative analysis of HaCaTs proliferation on microstructure membrane and flat membrane; ** *p* < 0.01, * *p* < 0.05.

As illustrated in Figure 8A (partial enlargement of Figure 8B), the cells on the membranes were evaluated via live/dead staining after HaCaTs were seeded and cultured for 3 days. HaCaTs were observed to be active at both the top and bottom of the microstructured nanofiber membrane microstructure by using confocal microscopy. Meanwhile, Figure 8C represents the proliferation rate of HaCaTs on microstructured membranes compared to flat membranes using the CCK-8 method. The study proved that the proliferation rates of HaCaTs were considerably higher on membranes with microstructure after 3 days of culture.

#### 3.3.3. HSFs Viability and Proliferation

**Figure 9:**
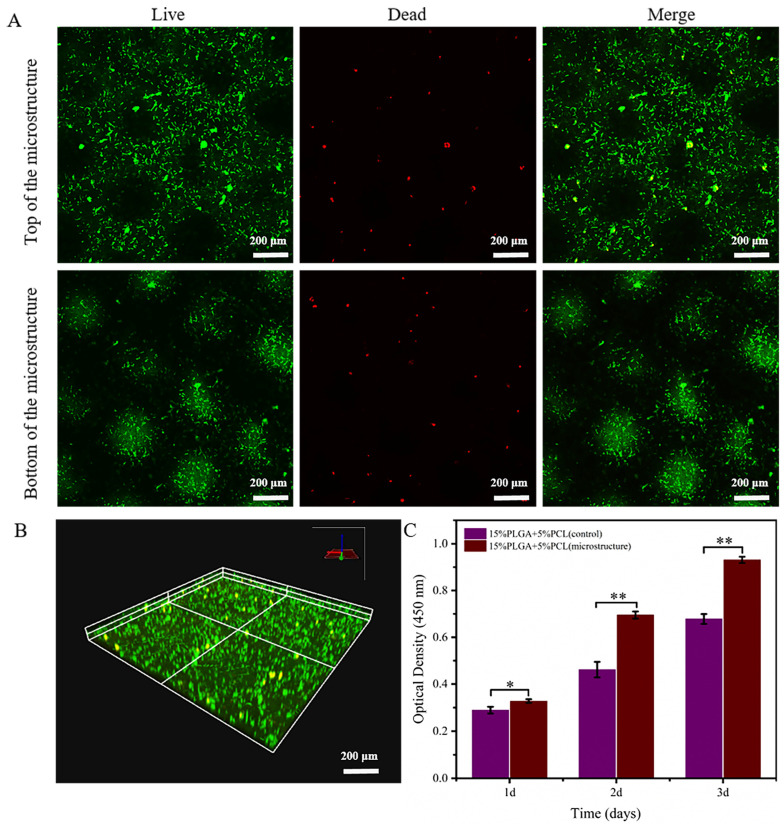
Evaluation of the viability and proliferation of HSFs on 15%PLGA + 5%PCL microstructure membranes. (**A**) Live/dead staining of HSFs. (**B**) Confocal images showing live/dead staining of HSFs on microstructure membranes. (**C**) Quantitative analysis of HSFs proliferation on microstructure membrane and flat membrane; ** *p* < 0.01, * *p* < 0.05.

The resulting images obtained by confocal microscopy in Figure 9A (a partial enlargement of Figure 9B) showed that fibroblasts were uniformly distributed on the nanofiber membranes after 3 days of culture. Furthermore, HSFs showed good cellular activity at both the top and bottom of the microstructure membrane.

Furthermore, the proliferation of HSFs on microstructure membranes and flat membranes was compared utilizing the CCK-8 method, as illustrated in Figure 9C. There was a statistically significant difference in the proliferation rate of fibroblasts on two different nanofiber membranes during the first day of culture. Moreover, the proliferation rate of HSFs on microstructure membranes was significantly higher than that on flat membranes during the second and third days.

## 4. Discussion

In previous studies, electrospun nanofibers were able to mimic the DEJ of human skin in terms of structure and function [44,45]. At present, PLGA has been widely used in skin tissue engineering due to its good biocompatibility and bioactivity [46]. However, the electrospun PLGA nanofiber membranes, which are subject to shrinkage in the cell culture environment, are unable to maintain the stability of the membrane dimensions for a long period of time [47,48]. We found that the addition of a certain concentration of PCL to the PLGA electrospinning solution can effectively improve the stability of PLGA nanofiber membranes. Therefore, we chose the PLGA-PCL membrane as the substrate to simulate the undulating structure of human skin DEJ.

The nanofiber membrane shrinkage test revealed that nanofiber membranes containing 15%PLGA and 15%PLGA + 3%PCL severely shrank in the cell culture environment. On the contrary, membranes containing 15%PLGA + 5%PCL and 15%PLGA + 7%PCL demonstrated good stability under these conditions. Additionally, analysis of the CCK-8 experiment demonstrated a higher proliferation rate of both HaCaTs and HSFs on the 15%PLGA + 5%PCL nanofiber membrane compared to the 15%PLGA + 7%PCL nanofiber membrane. The concentration of PCL can significantly enhance the mechanical strength and stability of PLGA nanofibers, but it can also result in reduced biological activity. Higher mechanical strength and stability of the membrane can facilitate the construction of precise microstructures on its surface. Thus, the nanofiber membranes consisting of 15%PLGA + 5%PCL, prepared through the micro-imprinting process with microstructures, possessed various benefits such as high mechanical strength, stability, biocompatibility, and bioactivity. The cell proliferation and live/dead tests of skin cells on 15%PLGA + 5%PCL microstructural membrane and flat membrane showed that the microstructural membrane was more conducive to cell adhesion and proliferation. 

Previous studies have utilized several methods to fabricate bionic skin tissue DEJ with microstructures, such as template-assisted electrospinning, laser etching, laser drilling, micro-milling, and photolithography [8,49,50,51]. Nevertheless, these aforementioned techniques can negatively impact the surface microform of the nanofiber membranes. Additionally, microstructures produced by laser etching and drilling techniques often possessed right-angled planes, which did not encourage favorable cell adhesion and proliferation [6]. The preparation of microstructure nanofibers through template-assisted electrospinning is constrained by the dimensions and shapes of the templates, and the removal of the templates may easily damage the surface microstructure, thus affecting its performance. In this study, a microstructure mold with high-precision 3D curvature was generated by 3D printing. A 3D curvature microstructure was successfully created on a 15%PLGA + 5%PCL membrane by imprinting microstructure mold. This advancement also enables the development of in vitro skin tissue models that better simulate natural skin. Previous studies have shown that microstructure size and shape on nanofiber membranes significantly affect cell adhesion, migration, proliferation, and differentiation [52]. Moreover, we will manufacture microstructural membranes of different sizes and shapes and seed skin cells for longer culture to further explore the influence of microstructures on the DEJ in future studies.

## 5. Conclusions

In this study, we selected a 15%PLGA + 5%PCL membrane with good comprehensive properties, which is suitable to be used as a substrate material to imitate the microstructure of skin DEJ through FTIR test, micro-morphology observation, dimensional stability, mechanical properties, and hydrophilicity. In addition, it was demonstrated that the microstructure generated by micro-imprinting nanofibers can promote cell proliferation and adhesion by seeding HaCaTs and HSFs in vitro and in detective cell proliferation and live/dead tests. We employ an innovative combination of 3D printing, electrospinning, and micro-imprinting techniques to produce nanofiber membranes that mimic the undulating structures of skin DEJ.

## Data Availability

The data that support the findings of this study are available from the corresponding author upon reasonable request.
